# Genome sequence of PSonyx, a singleton bacteriophage infecting *Corynebacterium glutamicum*

**DOI:** 10.1128/mra.01155-23

**Published:** 2024-01-18

**Authors:** Ombeline Rossier, Cécile Labarre, Anne Lopes, Monique Auberdiac, Kevin Tambosco, Daniel Delaruelle, Hakima Abes, Ana A. Arteni, Malika Ouldali, Laura Pieri, Ryan Afgoun, Leonor Anacleto, Nathan Beaure, Meyssa Beghdad, Nolwenn Bellom, Elsa Ben Hamou-Kuijpers, Aïda Boukamel, James Carron, Vincent Carta, Lauriane Castelneau, Zoe Chadaillac, Elsa Chaouat, Soline Desmat, Keylian Favel, Eva Gabillot, Melissa Gargar, Madeleine Gautheret, Esther Gilles, Claire Lager, Amandine Le Deit, Yoann Le vay, Laure Lemercier, Anastassiya Litvinov, Samir Moussi, Marion Prevot, Marion Rehala, Chloë Rodrigues, Ramatoulaye Sambe, Ashvini Srimoorthy, Tiroumagale M. Tillay, Cerise Verhoeven, Pauline Vittaz, Jacqueline Wu, Christophe Regeard

**Affiliations:** 1 Institute for Integrative Biology of the Cell (I2BC), CEA, CNRS, Université Paris-Saclay, Gif-sur-Yvette, France; 2 Faculté des Sciences d’Orsay, Université Paris-Saclay, Orsay, France; 3 Ecole Universitaire de Premier Cycle, Université Paris-Saclay, Orsay, France; Queens College Department of Biology, Ney York, USA

**Keywords:** virology, bacteriophages, genomes

## Abstract

PSonyx is a newly isolated phage that infects *Corynebacterium glutamicum*. This siphovirus was isolated from a French pond in the south of Paris by students from Paris-Saclay University. Its 80,277-bp singleton genome carries 136 protein-coding genes and 5 tRNAs.

## ANNOUNCEMENT

The Gram-positive bacterium *Corynebacterium glutamicum* is widely used for the mass production of glutamate and lysine (1). Although bacteriophages were previously reported to contaminate some industrial fermentation processes (2), so far only six phages infecting *C. glutamicum* were characterized at the morphological and genomic levels (3–8).

**Fig 1:**
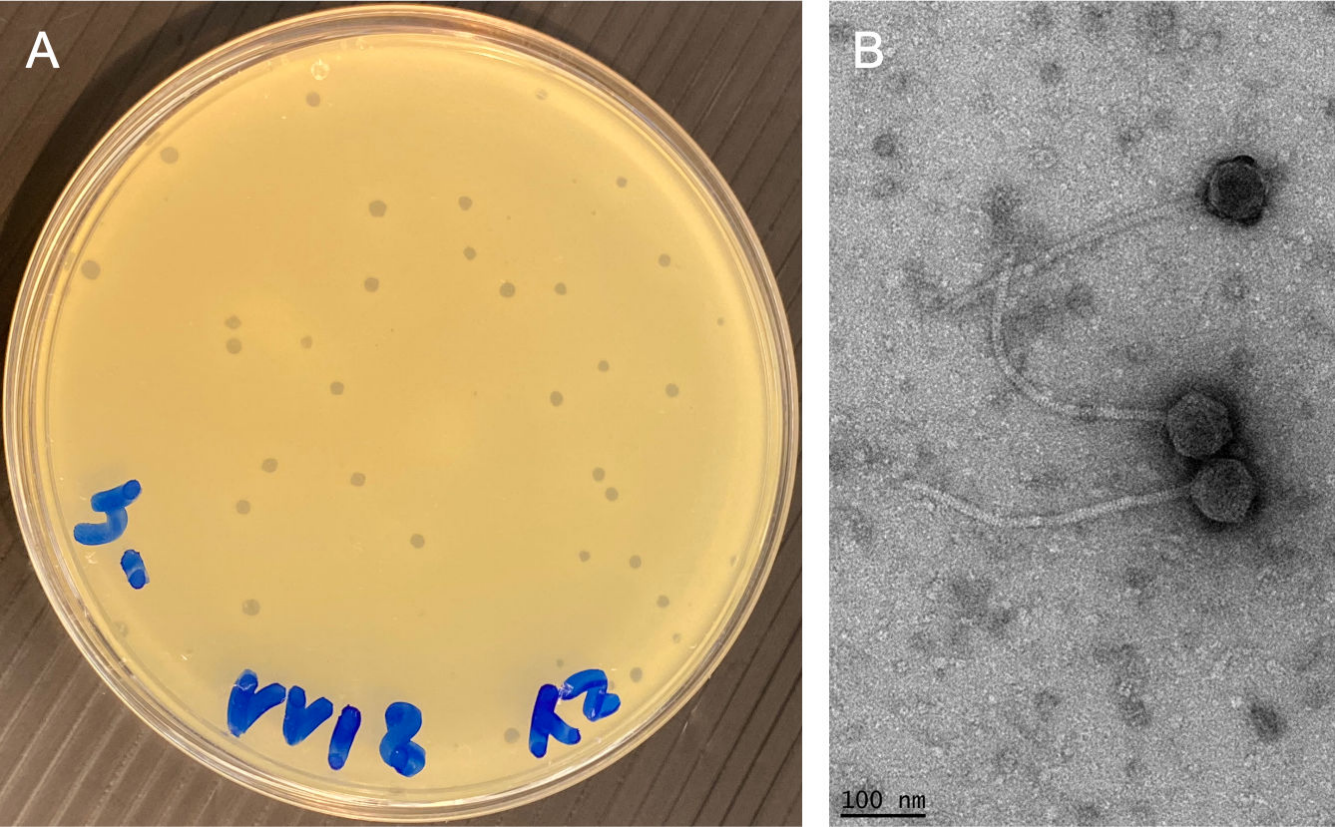
Characterization of siphovirus PSonyx. (**A**) Clear plaques observed in the LB agar overlay supplemented with *C. glutamicum* strain MB001 and 1 mM CaCl_2_ after 24 h at 30°C. (**B**) Negative staining of PSonyx phages with 2% wt/vol uranyl acetate observed with a Tecnai Spirit microscope operated at 100 kV (TFS) and equipped with a K2 Base 4kx4k direct electron detection camera (Ametek/Gatan). The magnification used was 19,800× with a pixel size of 2.5 Å at the level of the specimen. Bar: 100 nm.

Here, we report the complete genome of PSonyx, a bacteriophage that was isolated from pond water collected in Massy (48.72538 N, 2.27359 E), France. The sample was first passed through a 0.22-µm filter and then supplemented with LB (5 g/L NaCl; 5 g/L yeast extract; 10 g/L tryptone), 1 mM CaCl_2_, and *C. glutamicum* strain MB001, a derivative of the reference strain ATCC 13032 in which the prophages CGP1, CGP2, and CGP3 were deleted (8). After incubation for 24 h at 30°C, the filtered supernatant produced plaques when plated in the double agar layer in the presence of MB001 and incubated for 24 h at 30°C. The phage was purified through three rounds of plating using the procedures outlined in the SEA-PHAGES discovery guide (https://seaphagesphagediscoveryguide.helpdocsonline.com/). PSonyx showed clear plaques of approximately 1.5 mm diameter with irregular edges (Fig. 1). Transmission electron microscopy revealed a capsid with a diameter of 80 (±6) nm and a flexible tail with a length of 320 (±11) nm (*n* = 30) (Fig. 1).

Phage DNA was extracted from a high titer lysate using the PCI/SDS protocol (https://phagesdb.org/media/workflow/protocols/pdfs/PCI_SDS_DNA_Extraction_2.2013.pdf). A library was prepared using the NEB Ultra II kit and sequenced using an Illumina Miseq instrument (v3 reagents). *De novo* assembly of 133,928 single-end 150-bp reads (approximate coverage of 791) was performed with Newbler v2.9 as previously described (9), resulting in an 80,277-bp genome sequence, with a 53% G + C content and circularly permuted termini. Consed v29 (10) was used to check the completeness, accuracy, and termini.

Genome annotation identified 141 genes including 5 tRNAs with the following tools: DNA master v5.23.6 (http://cobamide2.bio.pitt.edu), Glimmer v3.02 (11), Genmark v2.5p (12), Aragorn v1.2.41 (13), and tRNAscan-SE v2.0 (14). Functional annotation was done with BlastP using NCBI nonredundant or actinobacteriophage databases (*e* value < 10^−3^) (15, 16), HHblits (prob > 70%) (17), and HHpred (18) using databases PDB_mmCIF70_18_Jun, Pfam-A_v36, Uniprot-Swissprot-viral70_3_Nov_2021, and NCBI_Conserved_Domain(CD)_v3.19. The analysis resulted in functional predictions for 42 protein-coding genes (31% of the total). Using TMHMM v2.0 (19) and SOSUI v1.11 (20), 13 proteins were predicted to localize to the membrane. All software were used with default settings.

The left third of the genome contains 30 genes that are transcribed forward and are likely involved in virion assembly and lysis. The encoded proteins include tail assembly chaperones using programmed translational frameshift, an endolysin, and a holin. The middle third of the genome is transcribed in reverse orientation. Although it contains 65% of the genes, most of them lack any known homologs, except for tRNAs and genes responsible for DNA replication/modifications. Finally, the last 19 genes are forward transcribed and include two intriguing long ORFs encoding proteins of 1,263 and 1,116 residues, respectively and both without homologs. The PSonyx genome does not share sufficient sequence homology with other genomes in the actinobacteriophage database and was therefore classified as a singleton.

## Data Availability

PSonyx is available at GenBank with accession No. OR521085.1 and Sequence Read Archive (SRA) No. SRX20630269.
